# Successful spinal anaesthesia for caesarean section in a patient with a dorsal root ganglion stimulation implant: A case report

**DOI:** 10.1016/j.crwh.2024.e00652

**Published:** 2024-10-09

**Authors:** Daniël P.C. van der Spek, Caroline D. van der Marel, Cecile C. de Vos, Frank J.P.M. Huygen, Maaike Dirckx

**Affiliations:** a Department of Anaesthesiology, Centre for Pain Medicine, Erasmus MC University Medical Centre, Rotterdam, the Netherlands; b Department of Anaesthesiology, Erasmus MC-Sophia Children's Hospital, Erasmus University Medical Centre, Rotterdam, the Netherlands

**Keywords:** Complex regional pain syndrome, Neurostimulation, Pregnancy, Caesarean, Case report

## Abstract

Neurostimulation, for example dorsal root ganglion stimulation (DRGS), is increasingly used for managing chronic pain, including among women of reproductive age. We present the case of a 33-year-old patient with complex regional pain syndrome (CRPS) implanted with DRGS who subsequently became pregnant twice. Both pregnancies resulted in the delivery of healthy newborns via caesarean section under successful spinal anaesthesia, with no (device) complications. This case highlights the special considerations for managing pregnant patients with neurostimulation implants, including the differences between DRGS implants and other neurostimulators in the context of neuraxial anaesthesia and the continued use of neurostimulation during pregnancy.

## Highlights

• This case highlights the special considerations for managing pregnant patients with neurostimulation implants.
• It highlights the differences between dorsal root ganglion stimulation implants and other neurostimulators in the context of neuraxial anaesthesia.
• The case highlights the continued use of neurostimulation during pregnancy.

## 1 Introduction

**Fig. 1:**
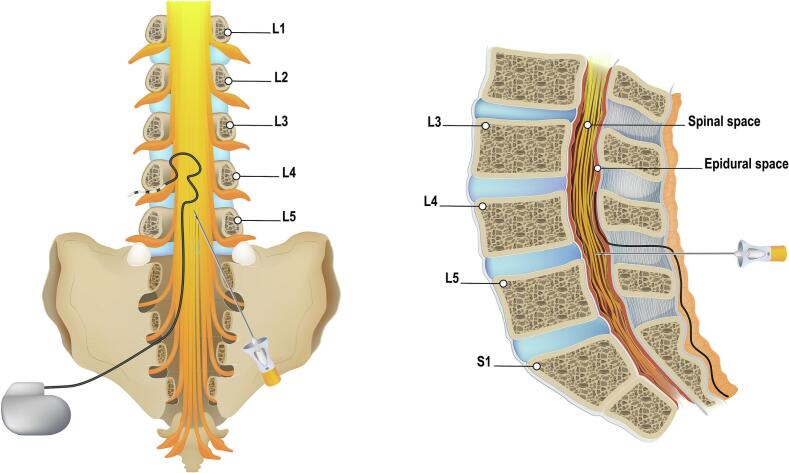
Overview of spinal anaesthesia in DRGS-implanted patients.

Neurostimulation is growing in popularity worldwide as a viable option for managing refractory symptoms in patients with chronic pain [1], including among women of reproductive age. As a result, pain physicians are increasingly likely to encounter female patients who either have an implanted neurostimulator or are considering one, and who may also desire pregnancy. Similarly, obstetricians and (obstetric) anaesthesiologists will also more frequently encounter patients with neurostimulators. For these implanted patients, additional precautions are necessary for future anaesthetic and obstetric management. These precautions include those related to the performance of neuraxial techniques [2] and the consideration of continued use of neurostimulation during pregnancy [3,4]. The precautions for neuraxial anaesthesia are particularly important for dorsal root ganglion stimulation (DRGS), as the electrode leads are placed in the epidural space at the dorsal root of the lower lumbar region (Fig. 1), which is also the typical site for neuraxial techniques in peripartum analgesia. In the literature, only 46 pregnancies are reported, with some illustrating the effect of the continued use of neurostimulation during pregnancy [[3], [4], [5], [6]]. However, these studies only involved spinal cord stimulation (SCS) and not DRGS-implanted patients. With this case study, we aim to highlight the special considerations for managing pregnant patients with neurostimulation implants, including the differences between DRGS implants and other neurostimulator for neuraxial anaesthesia and the continued use of neurostimulation during pregnancy.

This case report adheres to the applicable EQUATOR guidelines and written informed consent was obtained from the patient for its publication.

## 2 Case Presentation

At the age of 21, the patient had been involved in a scooter accident which resulted in CRPS-I according to the Budapest criteria. She had a normal BMI and no other medical conditions. Despite undergoing extensive physical therapy and trying multiple treatments, including analgesics, adjuvants, intravenous bisphosphonates, intravenous ketamine, and sympathetic blockades, the pain persisted, with a score of 7 on a numeric rating scale (NRS). Therefore, a DRG stimulator (Abbott Proclaim™) was implanted at the age of 27, targeting the left DRG at the level of L3 and L4, with the internal pulse generator (IPG) implanted in the left buttock. The painful area was completely covered, resulting in the gradual discontinuation of her pain medications, although some pain remained (NRS 4).

Nine months after implantation, the patient became pregnant with her first child. Extensive multidisciplinary consultations were held with the pain physician, (obstetric) anaesthesiologist, and obstetrician to discuss the different delivery options, anaesthetic strategies, and the continued use of neurostimulation during pregnancy. The patient expressed concerns about lead migration and difficulty positioning her knees during vaginal delivery, leading to a shared decision to proceed with a caesarean section. Although there were no excessive risk factors for complications with general anaesthesia, spinal anaesthesia (SA) was chosen based on the preference of both the patient and the team, as well as its favourable safety profile [7]. As the CRPS pain persisted during the pregnancy, she continued to use her DRGS until delivery. During this pregnancy, the patient reported increasing posture-dependent stimulation.

At gestational age (GA) 38 + 2, the patient was scheduled for a caesarean section. Prior to the SA, the DRGS was switched to surgery mode, and the radiographs were reviewed to increase safe and reliable performance of SA. Monitoring was performed by pulse oximeter, ECG, and automated non-invasive blood pressure measurements. Phenylephrine was used for prophylactic stabilization of blood pressure. With the patient in a sitting position, SA was performed at the L4–5 interspace, assessed through palpation. 2 mL bupi/sufentanyl (8 mg hyperbaric bupivacaine and 2 μg sufentanyl) was successfully injected intrathecally, resulting in an adequate sensory block (level Th4). A healthy baby girl was delivered by primary caesarean section 4 min after incision, using bipolar diathermy. The newborn weighed 3335 g and had Apgar scores of 9/10/10 after 1, 5, and 10 min, respectively.

No complications had been observed during the pregnancy. Postoperative testing of the device revealed no electromagnetic interference from the diathermy and confirmed normal functionality. However, one year after delivery, the stimulation suddenly ceased due to the breakage of two contact points on the L3 lead, necessitating lead replacement.

At the age of 32, the patient became pregnant again, expecting a boy. Remarkably, her CRPS pain improved significantly during this pregnancy, with an NRS score of 2, compared with 6 in the previous pregnancy. Once more, the DRGS was continued throughout the pregnancy. However, this pregnancy was complicated by gestational diabetes and at GA 38 + 3 a caesarean was urgently performed due to a suspected placental abruption. The procedure, monitoring and mixture used for the SA were similar to those of the previous caesarean. Adequate sensory block (level Th4) was uncomplicatedly achieved, performed at the L4–5 interspace, assessed through palpation. The newborn weighed 3850 g and had Apgar scores of 7/7/9. The gestational diabetes was effectively managed through dietary guidance and resolved after delivery. Table 1 summarizes both pregnancies.

**Table 1:** Summary of DRGS during pregnancy, delivery, and foetal outcomes.

	Pregnancy 1	Pregnancy 2
Continuation of DRGS during pregnancy	Yes	Yes
CRPS symptoms during pregnancy	No difference	Considerably improved
Delivery	Primary caesarean, SA L4–5	Early repeat caesarean for suspected placental abruption, SA L4–5
Continuation of DRGS peripartum	No	No
Foetal health	Healthy girl GA 38 + 2	Healthy boy GA 38 + 3
Apgar scores	9/10/10	7/9/9
Continuation of DRGS while breastfeeding	Yes	Yes
Maternal complications	None	Gestational diabetes
Technical complications/malfunctions	Postural dependency and two contact points broke on one of the leads one year after delivery	None

DRGS: dorsal root ganglion stimulation, CRPS: complex regional pain syndrome, SA: spinal anaesthesia, GA: gestational age.

Six months postpartum, the patient reported a slight increase in pain (NRS 3), despite the DRGS testing as fully functional. Both children were breastfed with the DRGS activated, with no reported difficulties in milk letdown. The patient was very satisfied with the shared-decision process, and the uncomplicated performance of the caesarean sections under SA.

## 3 Discussion

To our knowledge, no cases of DRGS-implanted patients undergoing SA for caesarean section have been documented in the literature. When considering the use of neurostimulation devices in patients of childbearing age, it is crucial to evaluate the potential implications for future anaesthetic and obstetric management.

### 3.1 Anaesthetic Management

In pregnant patients, neuraxial techniques are preferred over general anaesthesia due to their superior safety profile [7], and are advised to be performed below the entry level of the leads, regardless of the neurostimulation system [2]. However, DRGS differs from SCS because the leads are usually placed lower, around the level of L4 (Fig. 1), making it not always possible to perform neuraxial techniques below the leads. Epidural anaesthesia is not recommended due to the potential of epidural fibrosis, pre-existing scarring, and an increased risk of infection [2]. The epidural catheter could also theoretically damage, dislodge or become entangled with the leads. Although the potential for direct damage remains with SA, dislodgement of the lead is less likely when performed carefully. Alternatively, SA may be administered above the leads, although this approach carries a higher risk of nerve damage.

In the published reports of SCS during pregnancy, epidural anaesthesia was uncomplicatedly performed, but the leads were all inserted at L1 or higher [[8], [9], [10]]. When SA was performed, it was administered at least one lumbar level below the entry site of the leads [[4], [5], [6],[9], [10], [11], [12], [13]]. In the present case, after a careful review of the radiographs, SA was safely and reliably performed at the same level as the entry site of the DRG leads. It is recommended that recent radiographs be reviewed before performing SA to determine whether a median or paramedian approach is more appropriate and to assess the extent of the strain relief loops of the DRGS. Additionally, ultrasound guidance during the procedure may also be helpful [12].

### 3.2 Neurostimulation in Pregnancy

This case is the first to demonstrate the safe use of DRGS during pregnancy, with no observed foeto-maternal adverse effects. However, given the logical absence of randomized trials on this topic and the lack of cohort studies, it is not possible to provide high-level-evidence conclusions on the effects of neurostimulation during conception and pregnancy.

Patients known to be pregnant or planning for pregnancy shortly are not implanted, as placement requires fluoroscopic guidance. For implanted patients who become pregnant, some concerns have been expressed in the literature regarding the potential bidirectional effects of the continued use of neurostimulation during pregnancy [3]. Theoretically, the magnetic or electric field may affect foetal development [3]. However, given the low intensity generated by spinal neurostimulation, it is highly unlikely to cause fertility problems, miscarriages, or preterm labor [4]. Conversely, the reduction in pain and improvement in quality of life from neurostimulation could hypothetically increase fertility by reducing stress and potentially improve sexual functioning. In the present case, there seemed no fertility problems as the patient became pregnant promptly without requiring fertility treatment. In the published SCS cases (comprising 46 pregnancies) [[3], [4], [5], [6]], impaired foetal development has been described, although its occurrence is rare. There was one case of intrauterine growth restriction, six miscarriages in four women, and six preterm deliveries. Except for one, the miscarriages occurred in patients with previous clinical conditions or a history of complications [3,5]. Adversely, the alternative to neurostimulation primarily involves teratogenic medications. Moreover, lactation should not pose difficulties for neurostimulation patients and actually benefits newborns by reducing their exposure to (co-)analgesics through breast milk [14]. This is consistent with the case reported here. Finally, as the body mass and abdomen expand, technical issues like lead migration or breakage, stimulation interference, paraesthesia, or discomfort at the IPG may occur. Therefore, when considering neurostimulation for women of reproductive age, it is recommended to place the IPG in the buttock. This placement also reduces the risk of direct or electrocautery-related trauma during abdominal surgery [2], such as caesarean section. In the literature, only two device complications were reported: one hardware malfunction and one lead breakage [3]. The patient in the present case reported more posture-dependent stimulation, and on one lead, two contact points broke one year after the first pregnancy.

## 4 Conclusion

In conclusion, we presented the case of a DRGS-implanted chronic pain patient who underwent successful SA twice for caesarean section without any (device) complications. This report highlights the special considerations of (anaesthetic) management of patients with neurostimulation implants during pregnancy.

## References

[1] Knotkova H., Hamani C., Sivanesan E. (May 29 2021). Neuromodulation for chronic pain. Lancet.

[2] Orhurhu V., Hussain N., Karri J., Mariano E.R., Abd-Elsayed A. (Jun 2023). Perioperative and anesthetic considerations for the management of neuromodulation systems. Reg. Anesth. Pain Med..

[3] Camporeze B., Simm R., Maldaun M.V.C., Pires de Aguiar P.H. (Apr-Jun 2019). Spinal cord stimulation in pregnant patients: current perspectives of indications, complications, and results in pain control: a systematic review. Asian J. Neurosurg..

[4] Jozwiak M.J., Wu H. (Jan 2020). Complex regional pain syndrome management: an evaluation of the risks and benefits of spinal cord stimulator use in pregnancy. Pain Pract..

[5] Meier K., Glavind J., Milidou I., Sørensen J.C.H., Sandager P. (Jan 2023). Burst spinal cord stimulation in pregnancy: first clinical experiences. Neuromodulation.

[6] Zanfini B.A., De Martino S., Frassanito L. (May 23 2020). “Please mind the gap”: successful use of ultrasound-assisted spinal anesthesia for urgent cesarean section in a patient with implanted spinal cord stimulation system for giant chest wall arteriovenous malformation - a case report. BMC Anesthesiol..

[7] Delgado C., Ring L., Mushambi M.C. (Jun 2020). General anaesthesia in obstetrics. BJA Educ..

[8] Hanson J.L., Goodman E.J. (Jul 2006). Labor epidural placement in a woman with a cervical spinal cord stimulator. Int. J. Obstet. Anesth..

[9] Fedoroff I.C., Blackwell E., Malysh L., McDonald W.N., Boyd M. (Nov-Dec 2012). Spinal cord stimulation in pregnancy: a literature review. Neuromodulation.

[10] Young A.C., Lubenow T.R., Buvanendran A. (May-Jun 2015). The parturient with implanted spinal cord stimulator: management and review of the literature. Reg. Anesth. Pain Med..

[11] Ito S., Sugiura T., Azami T., Sasano H., Sobue K. (Feb 2013). Spinal cord stimulation for a woman with complex regional pain syndrome who wished to get pregnant. J. Anesth..

[12] Sommerfield D., Hu P., O’Keeffe D., McKeating A.K. (Jan 2010). Caesarean section in a parturient with a spinal cord stimulator. Int. J. Obstet. Anesth..

[13] Edelbroek C., Terheggen M. (Dec 2015). High-frequency spinal cord stimulation and pregnancy: a case report. Neuromodulation.

[14] Bernardini D.J., Pratt S.D., Takoudes T.C., Simopoulos T.T. (Oct 2010). Spinal cord stimulation and the pregnant patient-specific considerations for management: a case series and review of the literature. Neuromodulation.

